# Ultrasound-guided high-voltage pulsed radiofrequency versus standard-voltage pulsed radiofrequency in refractory chronic cervical radicular pain randomized clinical trial

**DOI:** 10.1007/s00540-025-03535-5

**Published:** 2025-07-05

**Authors:** Mohammed A. Alsaeid, Mohammed F. Algyar, Atef M. Mahmoud, Omar S. Farghaly, Ahmed G. Salah, Mina Maher Raouf

**Affiliations:** 1 Faculty of Medicine, Fayoum University, First Al Faiyum, Egypt; 2 Faculty of Medicine, Kafr Alshikh University, Kafr El-Shaikh, Egypt; 3 Faculty of Medicine, AL-Azhar University, Cairo, Egypt; 4 Faculty of Medicine, Minia University, Minia, Egypt

**Keywords:** High-voltage pulsed radiofrequency, Radicular pain, Visual analog scale, Neck disability index, Electrode temperature

## Abstract

**Purpose:**

To appraise the therapeutic impact of high-voltage pulsed radiofrequency (HVPRF) on the management of refractory unilateral cervical radicular pain.

**Methods:**

The study was conducted on 100 patients who had refractory chronic unilateral cervical radicular pain. Patients were divided into two equal groups (50 patients each): group (S): standard-voltage pulsed radiofrequency (SVPRF); and group (H): HVPRF. All patients received ultrasound-guided PRF on the affected cervical nerve root, either SVPRF in group (S) or HVPRF in group (H). The primary outcome was to assess the number of patients who experienced successful pain relief at 6 months’ post-intervention, which is defined as ≥ 50% reduction of visual analog scale (VAS) from pre-intervention level. Secondary outcome was to assess the impact of treatment on neck disability index (NDI) which was evaluated before treatment, 1, 3, and 6 months after the procedure.

**Results:**

VAS and NDI values were significantly reduced in group (H) in comparison to group (S) at all follow-ups. After 6 months, all patients in group (H) showed a clinically meaningful response with ≥ 50% decrease in VAS score. Conversely, no participants in group (S) attained a comparable decrease in their VAS scores.

**Conclusion:**

HVPRF could significantly reduce pain and functional disabilities compared to SVPRF up to 6 months after intervention in patients suffering from unilateral resistant cervical radiculopathy.

**IRB number**: Fayoum Faculty of Medicine Research Ethical Committee, approval no. R 427.

**Research registration number**: Registry for Clinical Trials (NCT05749185).

## Introduction

Chronic cervical radicular pain impacts approximately 35% of the population and is often caused by herniated discs resulting in chronic nerve root inflammation [1, 2]. Despite the availability of conservative treatments such as analgesics, physical therapy, and epidural steroid injections, many patients experience debilitating symptoms that impair daily functioning. Continuous radiofrequency (CRF) has been used for decades to manage chronic pain conditions such as trigeminal neuralgia (TN) and joint arthropathies [3], though the resulting high temperatures may potentially damage adjacent tissues [4]. Pulsed radiofrequency (PRF), a less destructive alternative maintaining temperatures below 42 °C, has demonstrated efficacy in alleviating neuropathic pain with a lower risk of tissue injury [5, 6]. Studies have supported PRF’s efficacy in treating cervical radicular pain [7, 8].

Radiofrequency waves, applied therapeutically since 1974, are delivered via needle electrodes that generate oscillating electrical fields, inducing ion movement and localized heating while maintaining safe tissue temperatures [9, 10]. The proposed mechanisms for PRF’s analgesic effects include modulation of descending inhibitory pathways, suppression of excitatory neurotransmitters, immune enhancement, reduction of inflammatory mediators, and induction of long-term synaptic depression [11–13]. Ultrasound guidance provides real-time, radiation-free imaging of critical structures, enhancing safety and precision [14].

Despite its promise, PRF outcomes vary, with some patients requiring repeated procedures, which increase both costs and dissatisfaction [7, 8, 15, 16]. The lack of long-term comparative data also limits its recommendation over alternatives such as cervical steroid injections or surgery [17]. Recent studies have focused on optimizing PRF efficacy through the use of higher intraoperative voltages. Teixeira and Sluijter demonstrated significant pain reduction using PRF at 60 V [18]. High-voltage PRF (HVPRF) maintains safe thermal levels while increasing voltage output, offering deep tissue penetration and potentially greater neuromodulator effects compared to standard-voltage PRF (SVPRF) [19].

The dorsal root ganglion (DRG) is central to chronic radiculopathy due to its vulnerability to mechanical compression and ectopic discharges [20]. Intermittent high-frequency electrical stimulation in the vicinity of the DRG selectively inhibits signal transmission in small unmyelinated C fibers, while sparing conduction in larger, myelinated fibers, contributing to targeted chronic pain modulation [21].

HVPRF represents a compelling option for managing chronic cervical radiculopathy, offering improved outcomes without additional risks. A recent meta-analysis of six randomized trials involving 423 candidates with neuropathic pain revealed that HVPRF provided significantly better pain relief at all follow-ups post-procedure (until 6 months) compared to SVPRF, with no increased procedure-related adverse events [22]. Moreover, HVPRF has been associated with improvements in sleep quality, emotional well-being, and reduced consumption and reliance on analgesics, marking its poly-faceted advantages [23]. Unlike SVPRF, which is typically limited to 45 V, HVPRF’s higher-voltage pulses maximize therapeutic effectiveness without exceeding the 42°C safety threshold [24].

Given the clinical need for more effective and durable pain control in refractory cervical radiculopathy, a direct comparison of HVPRF and SVPRF is warranted. The current study aimed to identify the optimal PRF parameters that enhance efficacy while minimizing risks for population with refractory unilateral cervical monoradiculopathy. We hypothesized that HVPRF would yield better results in terms of pain relief and disability reduction compared with SVPRF.

## Methods

The current study received approval from local Ethical Committee Council (approval no. R 427- Fayoum University) and registered on ClinicalTrials.gov with record number NCT05749185. This prospective, randomized, double-blind, single-center study took place at the Pain Management Department of Fayoum University Hospital from February 22, 2023 to March 12, 2024.

To ensure proper randomization and concealment, patients were divided into two groups using a computer-generated random number sequence with each group consisting of 50 patients. This computer-generated system was used to create randomization in blocks of five. Assignments were placed in sequentially sealed envelopes, which were opened prior to enrolling each case by a person who was unaware of the envelope contents and not part of the study team. All interventions were conducted by a single physician who was unaware of the specific type of PRF treatment being administered. Pre- and post-treatment data were collected by evaluators who were unaware of patients’ group assignments and were not part of the study team.

Inclusion criteria for the study were individuals aged between 20 and 70 years, of either sex, with an American Society of Anaesthesiologists (ASA) physical status of I or II [25], who presented with unilateral cervical radicular pain due to cervical disc prolapse affecting a single nerve root. Cervical radicular pain was diagnosed based on: A. clinical examination showing pain following a dermatomal pattern; B. cervical magnetic resonance imaging (MRI) revealing a unilateral single-level disc bulge affecting a single cervical nerve root; C. nerve conduction studies and electromyography findings indicating abnormalities consistent with proximal nerve involvement. It is important to note that these patients had not improved after 12 weeks of conservative treatments, including medications and physiotherapy. Prior to providing consent, all participants were thoroughly informed about the study’s nature, potential benefits, and possible adverse effects.

Exclusion criteria included individuals with axial neck pain without radicular symptoms, previous cervical spine surgery, cervical myelopathy, motor neurological deficits, radicular pain from multiple cervical discs prolapse levels, and pregnancy.

### Medical treatment details

#### A Pre-interventional

1. Naproxen sodium [220–500 mg twice daily (max 1,000–1250 mg/day] [26].
2. Pregabalin: 75 mg BID, flexible dosing up to 300 mg/day [27].
3. Gastroprotection**:** proton pump inhibitors (esomeprazole 20 mg PO once daily for 4 weeks) were administered on a case-by-case basis depending on gastrointestinal risk.
4. Physiotherapy**:** structured program: two to three sessions/week [28].
5. Manual therapy (including mobilizations and passive cervical lateral glides).
6. Global strengthening.

#### B Post-interventional

**Fig. 1:**
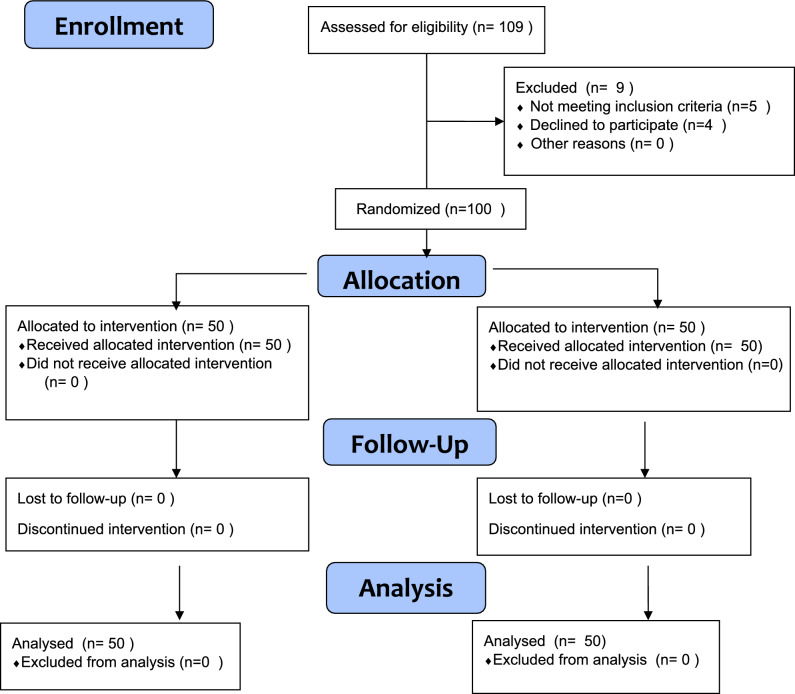
Demonstrates CONSORT flow

After intervention, all patients received the following protocol:

- Pregabalin one tablet (75 mg) twice daily.
- In case of breakthrough pain (flares), acetaminophen plus naproxen (multi-modal analgesia) was given [29].
- Physiotherapy was continued as such without change. Standardized physiotherapy protocols (intensity, exercises) were ensured across all study arms to isolate RF’s effects [30]. Adherence to conservative management protocols were closely monitored. Follow-ups were done via medication logs, patient diaries, physiotherapy session attendance, and checklist. Any modifications to the assigned conservative treatment during the study period were not allowed. Opioid use was prohibited even for unrelated issues (other opioid indications). In case of any deviation from this protocol, participants were excluded (CONSORT flowchart Fig. 1).

The study enrolled 100 patients who received 1 ml of 2% lidocaine during an ultrasound-guided diagnostic nerve block and reported at least 50% temporary reduction in pain. These participants were then divided into two equal groups (50 patients each) based on the type of radiofrequency procedure administered.

Two protocols were compared for PRF treatment in our study. Group (S) used a standard-voltage PRF (SVPRF), administering PRF at a fixed voltage of 45 V. Group (H) employed a high-voltage PRF (HVPRF) of 75 V. Both protocols involved two cycles of PRF application, with each cycle lasting 240 s and consisting of alternating periods of current application and deactivation. Pulse rate frequency was set to 2 Hertz (Hz), producing two active phases per second (20 ms, ms) of current application followed by 480 ms of no stimulation). Electrode tip temperature was carefully monitored to ensure it did not exceed 42°C in both groups. All interventions were performed by the same physician. Before the procedure, demographic data, disc herniation direction, location, laterality, and any adverse effects were recorded.

Pre-interventionally, every patient was examined, with tests including nerve conduction studies, MRI of the cervical spine, and clinical examinations to confirm diagnosis and the site of intervention. Visual analog scale (VAS) was utilized to determine pain severity; it is a linear scale with a length of 10 cm and a range from 0 (no pain) to 10 (extreme intolerable pain) [31]. Neck disability index (NDI) is a ten-item inventory which is used to assess the functional impact of pain on areas such as managing one’s own pain, lifting objects, reading, headaches, focus, job, driving, sleep, concentration, working, and activities. Each item is scored on a scale from 0 to 5, yielding a total sum ranging from 0 to 50. The neck disability Index (NDI) score is derived by summing the individual item scores. Functional impairment due to cervical disease is shown to be pronounced in cases with higher NDI scores [32].

### Therapeutic intervention

In this study, a modified ultrasound-guided technique was employed, based on the methods outlined by Nairouze et al. and Lee SH et al. [33, 34]. Each participant was placed in an oblique supine position with their head turned approximately 15 degrees to one side. A 12-MHz high-frequency linear transducer (Philips Ultrasound machine-USA) was employed, with a lubricating gel applied between the transducer and the skin to ensure clear imaging by eliminating air bubbles. The transducer was positioned transversely on the affected side of the neck, after the skin was aseptically prepared with 10% povidone iodine.

The process began by identifying the transverse process to determine the cervical spinal level. The unique shape of the C7 transverse process, characterized by its prominent, elongated posterior tubercle and short anterior tubercle, was used for identification. The acute anterior tubercle identified the C6 transverse process, while the “two-humped camel” appearance created by the anterior and posterior tubercles identified the C5 transverse process. A hypoechoic texture was observed at each cervical spinal level within the inter-tubercular groove of the transverse processes. Using real-time ultrasound guidance, a 22-gauge echogenic radiofrequency cannula with a curved tip (SMK Pole needle, 54 mm length, featuring a 4-mm active tip; Cotop International BV, Amsterdam, Netherlands) was carefully advanced toward the rear edge of the targeted nerve root. The in-plane approach was employed to insert the cannula until its tip was positioned adjacent to the nerve root, between it and the posterior tubercle of the cervical transverse process.

Color Doppler imaging was employed to verify the location of the vertebral artery around the nerve root, particularly at the C7 nerve root level, to avoid it during cannula advancement. A radiofrequency generator (Cosman G4—Cosman Medical, Burlington, MA) was then used to perform sensory and motor stimulation to confirm proper cannula placement. Patients were instructed to report sensations of dysesthesia, pain, or prolonged tingling in the affected areas during sensory stimulation at a frequency of 50 Hz and a voltage of 0.3 V as the cannula was gradually advanced. Motor response stimulation was carried out at 2 Hz with a 0.6 V to achieve the necessary motor stimulation for the targeted cervical nerve root. Ultimately, the tip of the cannula was positioned on the extraforaminal segment of the cervical nerve, beyond the DRG.

Patients’ vital signs were continuously monitored during the procedure and for 2 h afterward using non-invasive methods, including blood pressure, pulse oximetry, and electrocardiogram (ECG).

Primary outcome measure: Number of patients in each group who experienced successful pain relief at 6 months post-intervention, which is defined as ≥ 50% reduction of VAS compared to pre-intervention level.

Secondary outcomes**:** Impact of treatment on NDI before intervention and at 1, 3, and 6 months post-intervention, and occurrence of side effects. Overall, these protocols aimed to evaluate the efficacy and safety of different voltage settings in PRF for treatment of cervical radicular pain up to 6 months post-intervention.

### Sample size calculation

The required sample size was calculated using IBM© SPSS© Sample Power© version 3.0.1 (IBM© Corp., Armonk, NY, USA).

A previous study conducted by Lee et al. [35] reported that approximately 60% of patients undergoing conventional PRF for refractory chronic cervical radicular pain experienced successful pain relief at 6 months, which is defined as ≥ 50% reduction of VAS from pre-procedure level.

We targeted an absolute increase of 25% in the percentage of patients experiencing successful pain relief at 6 months with HVPRF (i.e., 85%). So, we estimated that 50 patients randomized into either study groups (total 100 patients) would achieve 81% power to detect statistical significance for this difference using a two-sided z-test with pooled variance when the type 1 error is set at 0.05.

### Statistical methods

Statistical analyses were conducted using IBM® SPSS® Statistics for Windows®, Version 27 (IBM Corp., Armonk, NY, 2020). The Shapiro–Wilk test was employed to assess the normality of numerical data distributions. Data conforming to a normal distribution were reported as mean and standard deviations, and comparisons between groups were performed using independent-samples *t*-tests. Categorical data are presented as counts and percentages and differences are compared with Chi-squared test.

Two-way repeated-measures analysis of variance was used to examine the effect of standard-voltage or high-voltage PRF on the change in VAS or NDI scores. Assumption of sphericity was tested using Mauchly test and Greenhouse–Geisser epsilon (ε). Greenhouse–Geisser correction was applied whenever this assumption was not met. Homogeneity of variance was examined using Levene test, while the assumption of normality was tested by examination of the Q–Q plots for standardized residuals versus theoretical quantiles. Tukey test was used for all post hoc pairwise comparisons.

*p* values < 0.05 are considered statistically significant.

## Results

The CONSORT flowchart is represented in Fig. 1. A total of 109 patients were enrolled in the study, with 9 patients were excluded either for not meeting the inclusion criteria or declining to participate. Fifty patients were assigned to each group, and all participants received the assigned intervention. Every patient was followed up for 6 months’ post-intervention. There was no loss to follow-up. The 6-month follow-up period facilitated successful tracking of all patients. It is important to note that patients with chronic radicular pain, particularly brachialgia, heavily rely on pain clinics due to the fluctuating nature of their condition. Brachialgia severely impacts quality of life and income, especially in developing countries. At the outset of the study, we thoroughly explained the potential benefits—such as pain relief and reduced functional disability—which motivated participants to adhere to the follow-up schedule. All included patients completed the study protocol without experiencing any adverse events.

Table 1 reports that demographic data (age, weight, sex), ASA physical status, involved disc level, direction of disc prolapses, and side of radiculopathy; there were no statistically significant differences between both groups (*p*-value > 0.05). As regards height and BMI, there were statistical differences between both groups (*p*-value < 0.05), but of no clinical significance.

**Table 1:** Baseline characteristics of both groups

Variable	Standard-voltage PRF (*N* = 50)	High-voltage PRF (*N* = 50)	*p*-value
Age (years)	49.0 ± 10.2	47.5 ± 7.6	0.427†
Weight (kg)	82.0 ± 6.2	81.8 ± 6.3	0.873†
Height (cm)	166.2 ± 10.0	172.1 ± 9.1	0.003†
BMI (kg/m^2^)	30.0 ± 4.5	27.9 ± 3.8	0.011†
Sex			0.155‡
Females	24 (48.0%)	17 (34.0%)	
Males	26 (52.0%)	33 (66.0%)	
ASA-PS			> 0.999‡
(I)	18 (36.0%)	18 (36.0%)	
(II)	32 (64.0%)	32 (64.0%)	
Involved disc level			0.717‡
C3/C4	9 (18.0%)	8 (16.0%)	
C4/C5	15 (30.0%)	16 (32.0%)	
C5/C6	9 (18.0%)	13 (26.0%)	
C6/C7	17 (34.0%)	13 (26.0%)	
Direction of prolapsed disk			0.890‡
Central	12 (24.0%)	10 (20.0%)	
Paracentral	20 (40.0%)	21 (42.0%)	
Foraminal	18 (36.0%)	19 (38.0%)	
Side of radiculopathy			0.317‡
Right	28 (56.0%)	23 (46.0%)	
Left	22 (44.0%)	27 (54.0%)	
Baseline VAS	86.1 ± 7.1	86.5 ± 7.0	0.746†
Baseline NDI	45.4 ± 2.8	45.7 ± 2.8	0.545†

Data are mean ± standard deviation or count (%)

^†^Independent-samples *t*-test

^‡^Pearson Chi-squared test

Table 2 shows that all patients in the HVPRF group had satisfactory response at 1 month, 3 months, and 6 months post-procedure as evidenced by a reduction of ≥ 50% in VAS and NDI compared to the baseline.

**Table 2:** Proportion of patients with satisfactory (≥ 50%) reduction in VAS and NDI compared to baseline

Variable	Time	Standard-voltage PRF (*N* = 50)	High-voltage PRF (*N* = 50)	*p*-value†
VAS reduction by ≥ 50% from baseline	1 month	50 (100.0%)	50 (100.0%)	NaN
	3 months	40 (80.0%)	50 (100.0%)	< 0.001
	6 months	0 (0.0%)	50 (100.0%)	< 0.001
NDI reduction by ≥ 50% from baseline	1 month	49 (98.0%)	50 (100.0%)	0.315
	3 months	28 (56.0%)	50 (100.0%)	< 0.001
	6 months	0 (0.0%)	50 (100.0%)	< 0.001

Data count (%)

^†^Pearson Chi-squared test

*Nan* not a number

In the SVPRF group, all patients had satisfactory response at 1 month as regards the reduction in VAS. This percentage dropped to 80% at 3 months, and at 6 months, none of the patients reported satisfactory reduction in VAS. As regards the NDI score, 98% of patients in this reported satisfactory reduction in NDI at 1 month. This percentage dropped to 56% at 3 months, and by 6 months none of the patients reported a satisfactory response.

The differences between the two groups as regards the percentage of patients with satisfactory reduction in the VAS or NDI were statistically significant at 3 and 6 months post-procedure (*p*-values < 0.001).

Notably, in the HVPRF group, the success rate was 100% at 1 month, 3 months, and 6 months post-procedure. However, this does not imply that all patients in this group were completely pain free. The success rate is defined as a ≥ 50% reduction in VAS compared to pre-intervention levels. While some patients in this group still reported some pain, particularly at the 6-month mark, their pain was reduced by more than 50% compared to the pre-intervention VAS.

As presented in Table 3, there were no statistically significant differences in baseline VAS and NDI scores between the two groups, with *p*-values of 0.746 and 0.545, respectively. Group (H) exhibited significant decrease in both VAS and NDI scores at all post-intervention time points when compared to group (S) (*p*-value =  < 0.001, 0.001, respectively).

**Table 3:** Main outcome measures

Variable	Time	Standard-voltage PRF (*N* = 50)		High-voltage PRF (*N* = 50)		Mean difference	95% CI		*p*-value†
		Mean	SD	Mean	SD		Lower	Upper	
VAS	Baseline	86.1	7.1	86.5	7.0	−0.5	−3.3	2.4	0.746
	one month	19.8	6.0	10.0	4.2	9.8	7.7	11.8	< 0.001
	three months	51.5	8.1	15.7	5.2	35.8	33.1	38.5	< 0.001
	Six months	80.3	6.5	20.4	6.2	59.8	57.3	62.4	< .001
NDI	Baseline	45.4	2.8	45.7	2.8	−0.3	−1.5	0.8	0.545
	one month	18.0	5.4	9.7	3.5	8.4	6.5	10.2	< 0.001
	three months	28.4	6.4	12.0	3.4	16.5	14.4	18.5	< 0.001
	six months	41.2	3.1	14.2	3.5	27.0	25.6	28.3	< 0.001

*95% CI* 95% confidence interval, *SD* standard deviation

^†^Independent-samples *t*-test

**Fig. 2:**
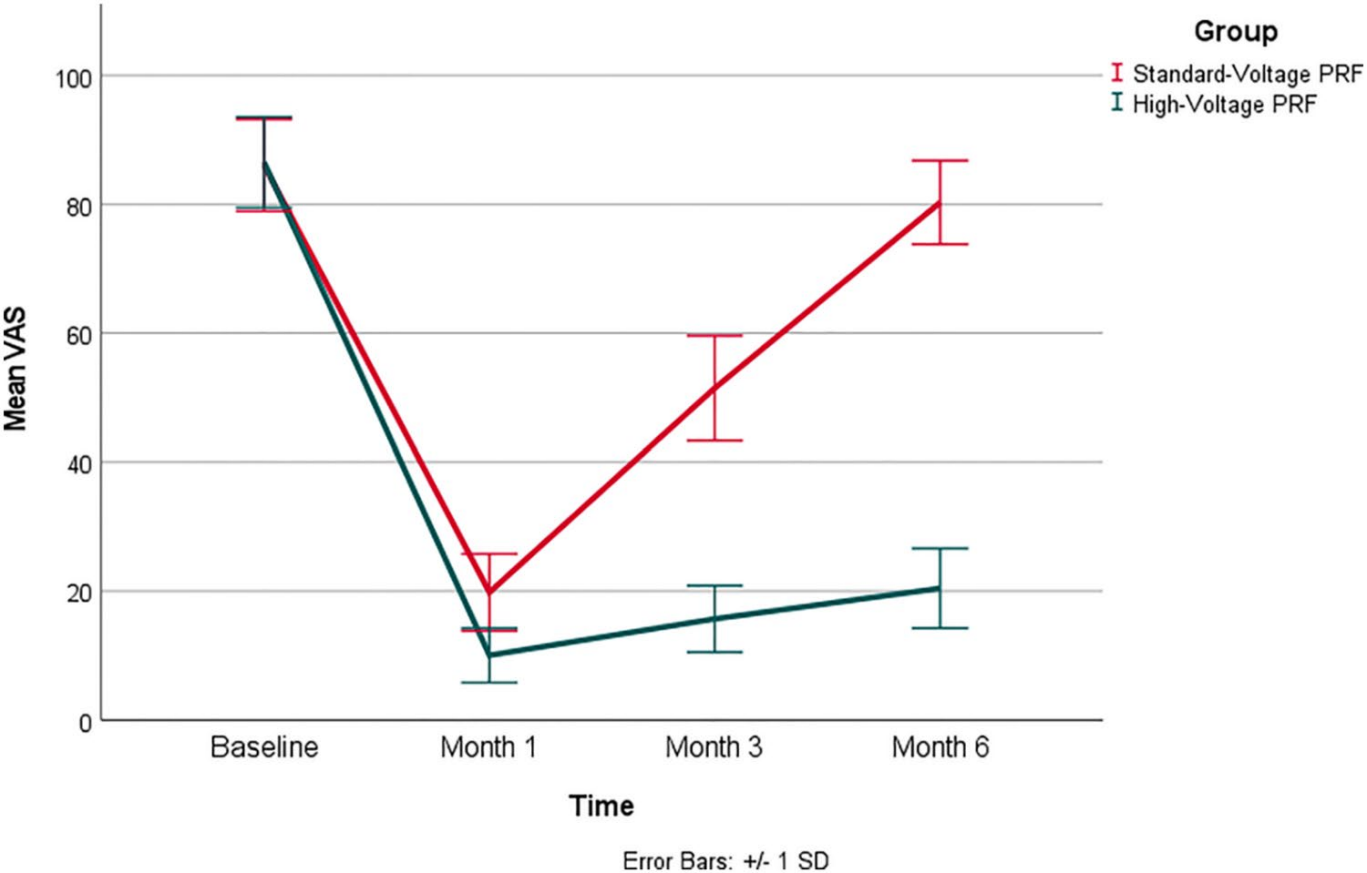
Shows mean VAS in both groups at pre-intervention, 1 month, 3 months and 6 months post-intervention. Error bars represent the standard deviation (SD)

**Fig. 3:**
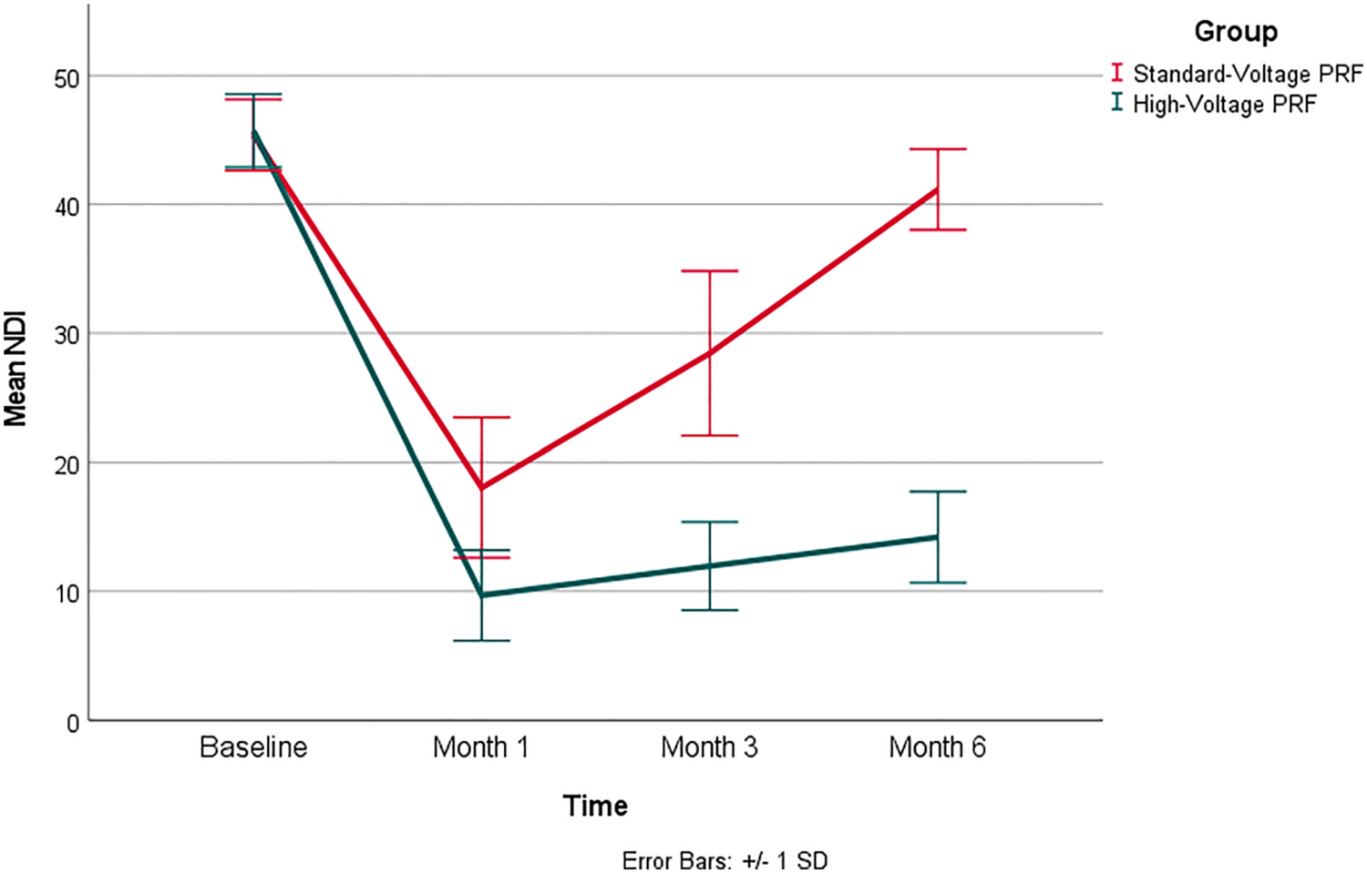
Shows mean NDI in both groups at preintervention, 1 month, 3 months and 6 months’ post-intervention. Error bars represent (SD)

Figures 2 and 3 show VAS and NDI scores in both groups. VAS and NDI scores were comparable at the pre-intervention time between both groups. Group (H) demonstrated significant reduction in both VAS and NDI scores compared to group (S) at all follow-ups.

## Discussion

The current study offers a novel and nuanced perspective by directly comparing the therapeutic efficacy of ultrasound-guided HVPRF and SVPRF in the population with unilateral, intractable cervical monoradiculopathy, a population that has been relatively underrepresented in previous randomized controlled trials (RCTs). To our knowledge, no prior RCT has directly compared these voltage modalities in this patient group under ultrasound guidance. Most existing studies have focused on the effects of PRF in conditions such as (TN) [36], and chronic lumbosacral neuropathic pain [37], reporting compelling results and a favorable safety profile.

Our study aligns with established biophysical principles, supporting the premise that higher voltage can enhance electric field penetration and cellular modulation around the target nerve without a proportional increase in adverse effects [38]. Given the similar pathophysiology of nerve root irritation and DRG sensitization across neuropathic pain syndromes, these benefits are likely applicable to cervical radiculopathy as well [39].

By assessing both pain and disability outcomes, this study addresses a significant gap in the literature. It also provides practical insights for optimizing PRF voltage parameters in this challenging clinical population. Further RCTs specifically targeting chronic cervical neuropathic pain are warranted to refine voltage settings and confirm these promising translational findings.

Many clinicians have conducted various clinical studies to optimize PRF’s effectiveness in pain management, adjusting parameters such as radiofrequency time, waveform length, temperature, and voltage settings [40, 41]. In our study, manual PRF mode of the radiofrequency generator was used to increase the output voltage in the HVPRF group, ranging from 65 to 80V depending on patient tolerance. This created strong electrical field in targeted tissues, while maintaining the temperature below 42 °C.

The current study found that patients who did not respond adequately to conservative treatments as drug therapy and physiotherapy for up to 12 weeks experienced significantly reduced pain scores and improved functional disability at all follow-ups with the HVPRF technique compared to SVPRF. Additionally, no adverse effects were observed in patients treated with either 42°C standard or HVPRF currents. Our findings suggest that the increased output voltage was safe, indicating that as long as the temperature remains at 42°C, higher output voltage is well tolerated and does not increase the risk of nerve injuries.

Many mechanisms have been proposed to explain HVPRF’s superiority over SVPRF. Sluijter et al. (2013) explain that PRF’s analgesic effect is related to the electric field strength, which is higher in HVPRF, potentially causing more effective neuromodulation without thermal damage [42]. HVPRF delivers higher voltage (e.g., 60–90 V) compared to SVPRF (typically 40–45 V), generating a stronger electric field around the targeted nerve. Mechanistically, the increased electric field may lead to more depolarization of neuronal membranes, which disrupts abnormal pain signal transmission more efficiently [22]. HVPRF has been shown to more effectively suppress pro-inflammatory cytokines (e.g., TNF-α, IL-6) and upregulate anti-inflammatory mediators (e.g., IL-10) exerting a broader neuro-immune modulatory effect. It also reduces the expression of pain-related genes (e.g., c-Fos, Nav1.7 sodium channels in dorsal horn neurons). HVPRF has been shown to interfere more effectively with action potential generation and ectopic neuronal firing, which underlies its superior analgesic efficacy. HVPRF appears to have a stronger inhibitory effect on spinal microglia and astrocytes, which play a key role in central sensitization and chronic pain maintenance [23]. These changes reduce neuro-inflammation, a key contributor to neuropathic pain. Some studies suggest that HVPRF promotes nerve repair by increasing brain-derived neurotrophic factor (BDNF) and Schwann cell activity [43]. HVPRF also appears to induce alterations in synaptic plasticity within pain pathways, leading to long-term depression of nociceptive transmission and sustained analgesia [44].

Supporting our findings, Sang Hoon Lee et al. investigated the efficacy of ultrasound-guided PRF guided in population with intractable cervical radiculopathy unresponsive to multiple epidural steroid injections, which is consistent with our results. They enrolled 49 patients who underwent ultrasound-guided PRF stimulation. The study demonstrated a significant decrease in the numeric rating scale, up to 6 months post-intervention. Additionally, there was a notable improvement in functional disability assessed by the NDI, at the 6-month follow-up. The authors suggested that ultrasound-guided PRF holds potential as a viable treatment modality for candidates with persistent cervical radiculopathy [35].

In 2018, Sang Gyu et al. conducted a meta-analysis to assess the impact of PRF in reducing cervical radicular pain. The analysis evaluated pain levels using VAS before and after PRF treatment on DRG. A total of 67 participants were analyzed across one randomized controlled trial, two prospective observational studies, and one retrospective investigation. Combined results showed a significant improvement in VAS following PRF at 2 weeks, 1 month, 3 months, and 6 months post-treatment. The meta-analysis concluded that PRF targeting the cervical DRG is an effective alternative when conservative treatments fail to relieve cervical radicular pain. Our study’s findings are consistent with these results [45].

In a randomized controlled trial by Simone Vigneri et al., the effectiveness of HVPRF of lumbar DRG was evaluated for persistent lumbosacral neuropathic pain. Forty-one candidates were assigned to two groups: the first one exhibited adhesiolysis followed by two cycles of HVPRF, while the second group underwent sham stimulation. At both 1- and 6-month follow-ups, HVPRF group reported significantly reduced lumbosacral pain compared to the control group. The authors concluded that HVPRF was effective in treating lumbosacral radicular pain. These results align with our findings, although our study focused on patients with chronic cervical radicular pain, targeting cervical nerve roots with HVPRF [46].

In a study by Luo Fang et al., a prospective, randomized, double-blind trial was conducted to evaluate the safety and efficacy of HVPRF compared to SVPRF in treating idiopathic TN using computed tomography guidance. Sixty patients were randomly assigned to receive CT-guided treatment targeting the Gasserian ganglion with either standard-voltage or high-voltage PRF between January and July 2012, with follow-up lasting 1 year. The findings demonstrated that CT-guided HVPRF is both a safe and effective treatment for TN. These results are consistent with our study, although we applied HVPRF to cervical nerve roots to treat chronic cervical radicular pain [19].

In 2017, *Luo Fang *et al. conducted a study to assess the effectiveness of HVPRF in treating refractory infraorbital neuralgia compared to SVPRF and found that HVPRF was effective in managing such neuralgia and attributed its enhanced efficacy to the stronger electric field in targeted tissues generated by the higher-voltage output. The intensity of electrical field is determined by dividing the square of the voltage by the resistance. Since resistance was comparable between the studied groups, they concluded that patients in the HVPRF group experienced stronger electric field effects in the targeted area of the infraorbital nerve due to the increased voltage output compared to the SVPRF group [36]. This could explain the mechanism by which patients in the HVPRF group in our study experienced better outcomes compared to the SVPRF group.

In 2021, Bo Wang et al. conducted a study comparing the effectiveness of PRF and HVPRF in treating acute herpes zoster neuralgia, focusing on thoracolumbar DRG. Sixty-four patients were followed for 12 weeks. The study found that HVPRF was more effective than SVPRF in relieving acute herpes zoster neuralgia. Our study’s findings are in line with these results [47].

Patient safety is paramount. No neurological complications were reported in the current study at all follow-up time points. Supporting this, Wang et al. (2024) conducted a meta-analysis of six RCTs (*n* = 423) comparing HVPRF and SVPRF in chronic neuropathic pain of various settings. They found that HVPRF provided superior pain relief up to 180 days post-intervention without increasing any neurological risk [22]. Wang et al. 2017 reported no neurological complications at all follow-ups in their clinical trial of pulsed radiofrequency treatment for chronic cervical radiculopathy [48]. This explicitly documents the credibility and safety of PRF for chronic neuropathic pain.

### Study limitations

Our study has several limitations. First, the 6-month follow-up period may not fully capture the long-term efficacy of the treatment. Extending the follow-up to 9 months or longer could offer more thorough understanding of the intervention’s sustained effectiveness. Secondly, patient recruitment was limited to a single hospital, and the sample size was relatively small. A multi-center study would improve the generalizability and strength of the findings. Another limitation is the potential influence of pre-interventional psychological factors on patient-reported outcomes. Although NDI includes components related to concentration and emotional well-being, it remains a composite measure that may not fully reflect the full spectrum or severity of psychological conditions such as anxiety or depression. Consequently, similar NDI scores between groups may not imply equivalence in psychological status. Future studies incorporating validated psychiatric assessment tools—such as the Patient Health Questionnaire-9 (PHQ-9) or Beck Depression Inventory (BDI)—are warranted to better evaluate the impact of psychological comorbidities on treatment outcomes.

## Conclusion

Application of HVPRF for cervical nerve roots significantly reduces pain intensity and improves quality of life in patients with chronic cervical radiculopathy refractory to conservative modalities, which could be maintained for up to 6 months’ post-intervention.

## Data Availability

Data avaliable upon request.
